# Simultaneous quantification of actin monomer and filament dynamics with modeling-assisted analysis of photoactivation

**DOI:** 10.1242/jcs.194670

**Published:** 2016-12-15

**Authors:** Maryna Kapustina, Tracy-Ann Read, Eric A. Vitriol

**Affiliations:** 1 Department of Cell Biology and Physiology, School of Medicine, University of North Carolina, Chapel Hill, NC 27599, USA; 2 Department of Anatomy and Cell Biology, University of Florida, Gainesville, FL 32610, USA

**Keywords:** Actin, Modeling, Photoactivation, Cofilin, Axonal cytoskeleton

## Abstract

Photoactivation allows one to pulse-label molecules and obtain quantitative data about their behavior. We have devised a new modeling-based analysis for photoactivatable actin experiments that simultaneously measures properties of monomeric and filamentous actin in a three-dimensional cellular environment. We use this method to determine differences in the dynamic behavior of β- and γ-actin isoforms, showing that both inhabit filaments that depolymerize at equal rates but that β-actin exists in a higher monomer-to-filament ratio. We also demonstrate that cofilin (cofilin 1) equally accelerates depolymerization of filaments made from both isoforms, but is only required to maintain the β-actin monomer pool. Finally, we used modeling-based analysis to assess actin dynamics in axon-like projections of differentiating neuroblastoma cells, showing that the actin monomer concentration is significantly depleted as the axon develops. Importantly, these results would not have been obtained using traditional half-time analysis. Given that parameters of the publicly available modeling platform can be adjusted to suit the experimental system of the user, this method can easily be used to quantify actin dynamics in many different cell types and subcellular compartments.

## Abstract (precis)

**Summary:** We have devised a new modeling-based analysis for photoactivatable actin experiments that accurately measures multiple parameters of actin dynamics, providing more detailed results than those obtained using traditional half-time analysis.

## INTRODUCTION

Photoactivation is a powerful tool to study the dynamic nature of proteins given that it allows molecules from a specific subcellular compartment to be identified and followed over time. With the advent of genetically encoded photoactivatable and photoconvertable fluorescent proteins (Adam et al., 2014; Lippincott-Schwartz and Patterson, 2009; Patterson and Lippincott-Schwartz, 2002, 2004), and the commercial development of user-friendly confocal microscope setups, photoactivation has become more accessible than ever. Studies of the actin cytoskeleton, in which assembly from cytoplasmic pools of monomers (G-actin) into filaments (F-actin) is highly organized in space and time, have greatly benefited from this technique. In fact, actin was one of the first proteins to be used in photoactivation experiments, where pulse-labeling a subset of the lamellipodia demonstrated rapid turnover of actin filaments through assembly at the leading edge, retrograde flow and disassembly at the rear (Theriot and Mitchison, 1991). Since then, photoactivation has proven useful in understanding how the actin cytoskeleton is dynamically regulated during cell migration, in response to extracellular signals, during *Listeria* motility, in organizing the cellular cortex and in regulating the neuronal synapse (Abella et al., 2016; Burnette et al., 2011; Fritzsche et al., 2013; Frost et al., 2010; Higashida et al., 2013; Honkura et al., 2008; Kiuchi et al., 2011, 2007; Lai et al., 2008; Vitriol et al., 2015).

However, there is now an increased need for computational tools to extract more detailed and accurate information that goes beyond the traditional calculation of half-times (*t*_1/2_) and immobile fractions that are commonly done with fluorescence recovery after photobleaching (FRAP) and photoactivation experiments. Some strategies have been developed to meet this demand. For example, one method tailored specifically to actin called sequential fluorescence decay after photoactivation (sFDAP) uses sequential photoactivation in a single region of the cell in order to obtain information about the local G-actin concentration and how it changes with respect to extracellular stimuli (Higashida et al., 2013; Kiuchi et al., 2011). However, one problem with deriving accurate information from photoactivation or photobleaching experiments is that the analysis often fails to account for all of the complex real-world details of an experiment (Carrero et al., 2003; Halavatyi et al., 2010; Sprague et al., 2004; Tardy et al., 1995). These include, but are not limited to: non-isotropic diffusion due to specific cell morphology, a substantial loss of information due to the delay between photoactivation and imaging recording, and the fact that photoactivation is non-instantaneous. To deal with these shortcomings, mathematical modeling can be a particularly useful tool for understanding the experimental system and overcoming its limitations.

Here, we describe a computational method that compensates for the potential pitfalls that often occur during analysis of photoactivation experiments and allows for the calculation of accurate subcellular information about actin diffusion (Vitriol et al., 2015), the proportion of actin monomers to filaments, and F-actin turnover rates. The method is based on the freely available Virtual Cell (http://vcell.org) modeling platform, which permits incorporation of spatial and biochemical details in a three-dimensional system (Slepchenko and Loew, 2010). It works by first simulating the photoactivation of actin in a custom Virtual Cell environment that matches the geometry of the actual cells the experiments are being performed in. The conditions (G-actin concentration, F-actin depolymerization rates, etc.) are varied in small increments to create a library of hundreds of potential outcomes for that particular photoactivation experiment. That data is then used to fit experimental photoactivation decay curves. Our models are publically accessible on the Virtual Cell server and can be adapted to fit any experimental system of the user. We believe that our modeling-based approach will serve as an excellent tool to quantify the local dynamics of actin in a large variety of experimental systems and provide more detailed information than can be obtained by current methods of analysis.

## RESULTS

**Fig. 1.**
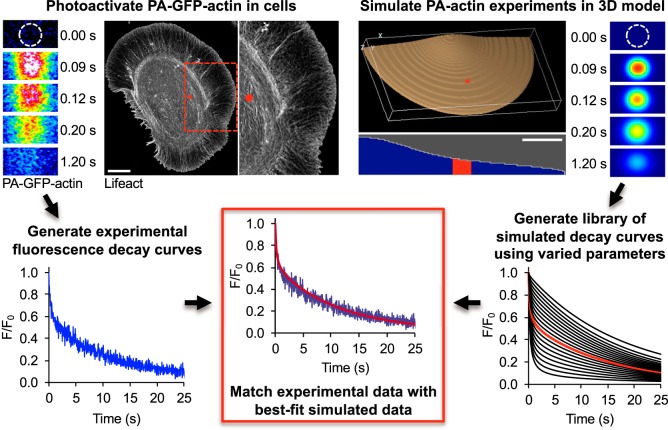
**Schematic of MAAP.** Actin is photoactivated in live cells in a 2-μm circular region (red circle) 20 μm from the leading edge (upper left). Scale bar: 10 μm. This generates a fluorescence decay curve (bottom left). We also simulate actin photoactivation using Virtual Cell (upper right). Shown are a three-dimensional image of the cell geometry used for modeling and a two-dimensional side view. The photoactivated area is highlighted in red. To expedite computation, modeling was performed in a half-cell geometry. Scale bar: 5 μm. The right-hand panels in this section show images of a simulated PA-GFP–actin experiment. The model is used to generate a library of simulations that represent potential outcomes of photoactivation experiments (bottom right). Experimental data is then matched with the best-fit computational data by determining the match that has the lowest root-mean-square deviation (bottom center).

We devised a mathematical model to perform *in silico* simulations of fluorescence decay after photoactivation. Using different combinations of values for molecular concentrations and reaction rates, this model could then generate a library of potential outcomes of a given photoactivation experiment. This library is then used to fit experimental data and describe the observed behavior of actin that occurred during the actual experiment. The model was built using the Virtual Cell platform. Virtual Cell allows for equation-based simulations to be performed in a three-dimensional environment where reactions can be spatially contained (Slepchenko and Loew, 2010). In our Virtual Cell model, we constructed a cellular geometry which mimics the round morphology of the cath.A-differentiated (CAD) neuroblastoma cells used in our experiments (Fig. 1; see Materials and Methods for details). In the model, as in the experiments, actin is locally photoactivated and its concentration can be followed over time, resulting in decay curves similar to those derived from cells imaged on the microscope (Fig. 1). We have named this methodology modeling-assisted analysis of photoactivation (MAAP).

**Fig. 2.**
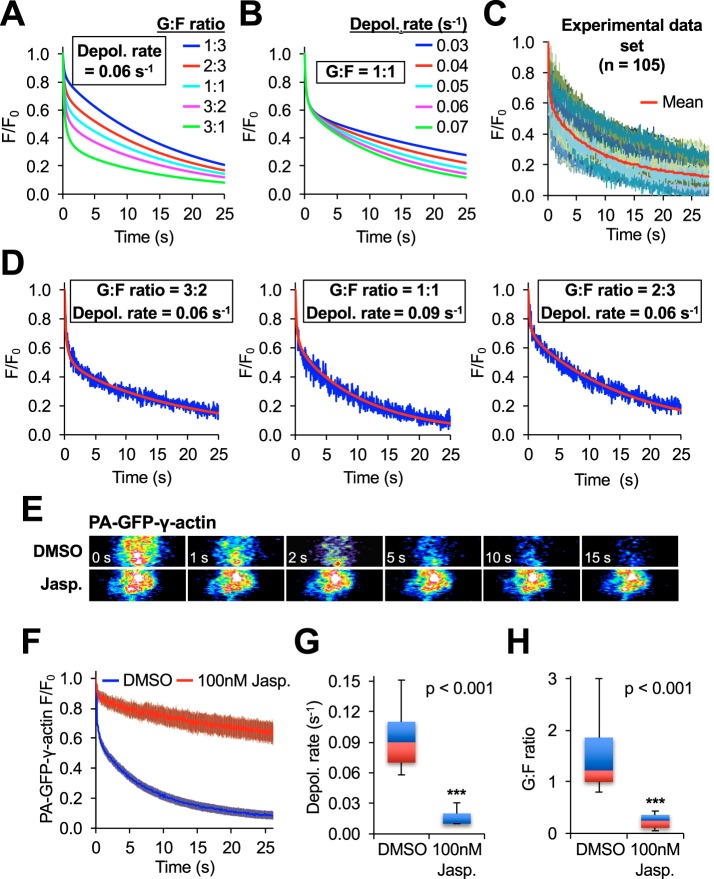
**MAAP of PA-GFP–actin fluorescence decay curves allows for accurate, simultaneous calculation of the G-actin:F-actin ratio and F-actin disassembly rates.** (A) Simulated data for PA-GFP–actin showing the effect that changing the local G-actin:F-actin (G:F) ratio has on the fluorescence decay curves. All data was simulated with an F-actin depolymerization rate (Depol. rate) of 0.06/s. (B) Simulated data for PA-GFP–actin showing the effect that changing the depolymerization rate has on the fluorescence decay curves. All data was simulated with a G:F ratio of 1:1. (C) A data set of 105 PA-GFP–actin fluorescence decay curves and their mean (red). (D) Examples of individual curves (blue) from E matched with the simulated data with the combination of G:F ratio and depolymerization rate that gave the best fit (red). (E) Representative photoactivation images from live cells that were pre-treated with DMSO or 100 nM Jasplakinolide for 15 min. (F) Mean decay curves for DMSO (*n*=40) and Jasplakinolide-treated (*n*=20) data sets. Error bars are 95% confidence intervals. (G) Box-and-whisker plot showing the distribution of calculated depolymerization rates from individual experiments. (H) Box-and-whisker plot showing the distribution of calculated G:F ratios from individual experiments. Box-and-whisker plots denote the 95th (top whisker), 75th (top edge of box), 25th (bottom edge of box) and 5th (bottom whisker) percentiles, and the median (bold line in box). *P*-values are from a two-tailed Student's *t*-test.

Working under the assumption that G-actin was freely diffusing and F-actin was stationary during the time scale of our experiments, the two prevailing parameters that had dominant effects on decay curves in our model of actin photoactivation were: the G-actin:F-actin ratio and the filament depolymerization rate. We used the model to calculate the decay curves for a wide range of G-actin:F-actin ratios (from 1:9 to 9:1 with 0.5 steps) and F-actin depolymerization rates (from 0.0 s^−1^ to 0.20 s^−1^ with 0.01 s^−1^ steps) to generate a library with 380 different photoactivation outcome scenarios. Examples of fluorescence decay curves for different parameter sets are presented in Fig. 2D. Freely diffusing monomeric actin is responsible for the initial rapid loss of fluorescence; the G-actin:F-actin ratio determines how much fluorescence is lost during this period (Fig. 2A). After actin monomers diffuse away from the photoactivated region, the much slower decay of fluorescence is determined by the filament depolymerization rate (Fig. 2B).

For simplicity, we considered that all forms of G-actin (free G-actin and G-actin in complex with monomer-binding proteins) had the same diffusion and average polymerization rates. To further simplify the model, we assumed that the F-actin concentration and G-actin diffusion were constant during the simulation. To test whether we were correct in our decision to exclude F-actin oscillations from our analysis, we normalized the intensity of photoactivatable GFP (PA-GFP)–actin data against the intensity of Lifeact–mRuby, which was recorded simultaneously during the experiment. Lifeact is a small peptide that reversibly binds F-actin and is commonly used as an F-actin marker for live-cell imaging (Riedl et al., 2008). Normalizing the PA-GFP–actin data against Lifeact fluorescence intensity had no effect on the resultant fluorescence decay curves (Table S1), demonstrating that fluctuations and movement of F-actin through the photoactivated region did not substantially alter the outcome of the experiments and could therefore safely be ignored for our purposes.

In previous studies, we used a diffusion-only (without polymerization) iteration of MAAP to determine the rate of free diffusion for G-actin in CAD cells (Vitriol et al., 2015). This analysis was performed using actin point mutants that allowed the actin to remain soluble but prevented it from polymerizing. The fluorescence decay at the photoactivated region of interest (PA ROI) was rapid with these point mutants, with almost 80% of the initial fluorescence lost in 1.5 s (Fig. 1B). We found the average diffusion rate of monomeric actin to be 3 µm^2^/s (Vitriol et al., 2015). However, a more detailed analysis revealed that the diffusion rate of individual experiments is distributed between 2 µm^2^/s and 5 µm^2^/s (Fig. S1A). To verify that the same diffusion rate calculated for monomeric point mutants also applies to wild-type (WT) PA-GFP–actin, we compared the fluorescence decay curves of point mutants with the initial section of WT PA-GFP–actin decay curves. To do this, we assumed that the fluorescence that remains in the photoactivated ROI at 2.5 s is coming from F-actin and could be treated as an immobile fraction during the first 1.5 s of recording. After subtraction of the immobile fractions, the average decay dynamics for both WT and mutant PA-GFP–actin fluorescence was essentially identical (Fig. S1B). Next, we tested how the distribution of G-actin diffusion rates affected fluorescence decay dynamics on a longer time scale, when 50% of actin molecules are contained in filaments. The computational analysis showed that the difference between fluorescence decay curves with diffusion rates of 3 µm^2^/s, 5 µm^2^/s, and even 10 µm^2^/s is negligible and does not affect our fitting accuracy (Fig. S1C), so that assuming a single diffusion rate of 3 µm^2^/s is sufficient for our analysis.

To accurately calculate the rate of free diffusion, it is important to account for the loss of fluorescence occurring throughout photoactivation and during the time delay between the end of photoactivation and the beginning of the next recorded frame (Vitriol et al., 2015; Fig. S1D). To determine how much fluorescence in a freely diffusive system can be lost during the delay between photoactivation and recording of the next image sequence, we generated simulated data using different diffusion rates and delays after photoactivation. Even for a slowly diffusing molecule of 3 μm^2^/s, a delay of just 100 ms can result in a 30% loss of fluorescence before imaging begins. For something diffusing at 20 μm^2^/s, a 30% fluorescence loss happens with only a 20-ms delay and fluorescence loss is over 60% if the delay is 100 ms (Fig. S1F). Delays of this length are quite realistic for most commonly used microscopy setups. Therefore, we proceeded to verify how substantial this delay is for fitting fluorescence decay curves of WT actin that has photoactivated molecules in both G- and F-actin forms. We assumed that the fluorescence intensity value at 0.035 s or 0.235 s is the first recorded frame being considered as the beginning of decay curve in two separate experimental setups. The difference in decay dynamics for the different delay times were quite substantial, and greatest in the system with the slower depolymerization rate (Fig. S1G). Therefore, accurate analysis of actin photoactivation curves must consider this time delay. By using MAAP, we are able to properly compensate for these factors.

**Fig. 3.**
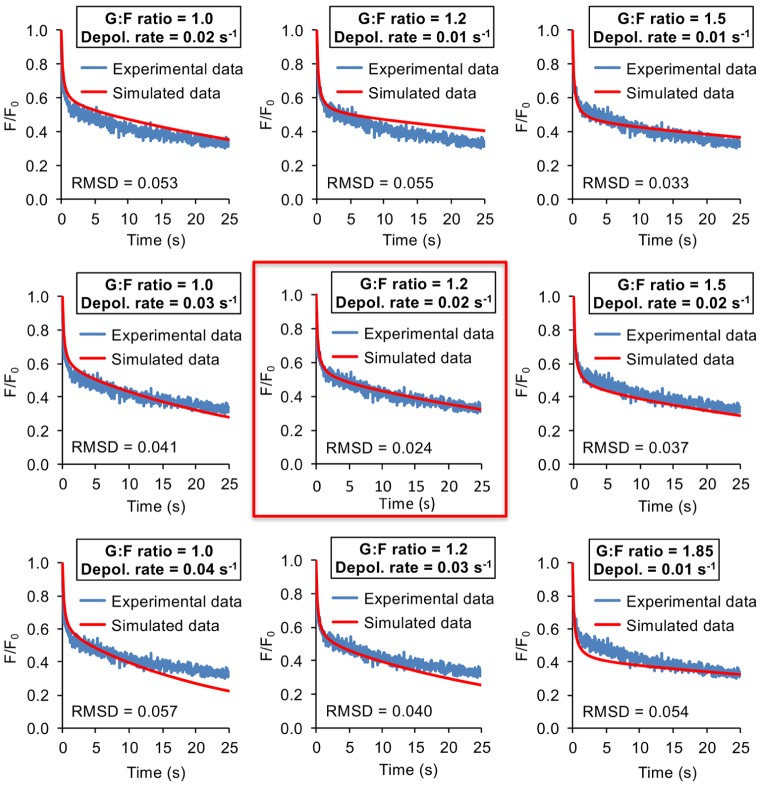
**Accuracy of MAAP fitting of experimental data.** Line graphs showing the nine closest MAAP fits to a single experiment. The fit in the middle, outlined in red, is the one chosen by MAAP because it had the lowest root-mean-square deviation (RMSD).

In FRAP and photoactivation studies, there is often variability between individual experiments. Fig. 2C shows a data set of 105 PA-GFP–actin decay curves obtained from 45 unique cells. It is apparent that individual experiments display a marked heterogeneity and present a range of G-actin:F-actin ratios and depolymerization rates despite being performed in equivalent subcellular regions and in cells of similar morphology. Even experiments performed in the same cell often exhibited different fluorescence decay dynamics (Fig. S2A). Thus, we decided to analyze each fluorescence decay curve individually rather than perform an analysis on the mean curve of the data set. The best-fit computational curve was chosen by determining which had lowest root-mean-square deviation (RMSD) to the experimental curve. We were able to obtain excellent fits for individual experiments (see Fig. 2D for examples of fits using different parameters; see Fig. 3 for an example of fitting accuracy of a single experimental curve; see Table S2 for an analysis of all data included in this study). Furthermore, a detailed sensitivity analysis revealed how the fluorescence signal in the PA ROI responds to small changes in the G-actin:F-actin ratio and the depolymerization rate after actin photoactivation (Figs S3 and S4, detailed in Materials and Methods). We found that the highest sensitivity to F-actin depolymerization rate was when high concentration of F-actin had a slow depolymerization rate, and the maximum sensitivity of the G-actin:F-actin was around *t*=2 s, with higher G-actin:F-actin ratios having bigger sensitivity. Ultimately, this means that within the range of values found in our experimental data, MAAP has an excellent ability to differentiate between small differences in actin dynamics and provide an accurate fit for the data.

To verify that we were able to simultaneously measure G-actin diffusion and F-actin depolymerization, we used the actin drug Jasplakinolide (Bubb et al., 1994). Jasplakinolide stabilizes actin filaments at intermediate concentrations but does not prevent polymerization. The net result is that, at the correct concentration, depolymerization nearly comes to a halt and all of the available G-actin is converted into F-actin. Thus, both the G-actin:F-actin ratio and the depolymerization rate should be substantially reduced. As expected, a 15-min pre-treatment with 100 nM Jasplakinolide did cause a severe reduction in both the observed depolymerization rates and G-actin:F-actin ratio (Fig. 2E–H). The reduction of the G-actin:F-actin ratio from 1.51±0.23 to 0.24±0.07 (mean±95% c.i.; *P*=2.3×10^−10^), or an 86% average drop in available actin monomers, is consistent with previously reported experiments using Jasplakinolide where G-actin concentration was measured using sFDAP analysis (Kiuchi et al., 2011).

**Fig. 4.**
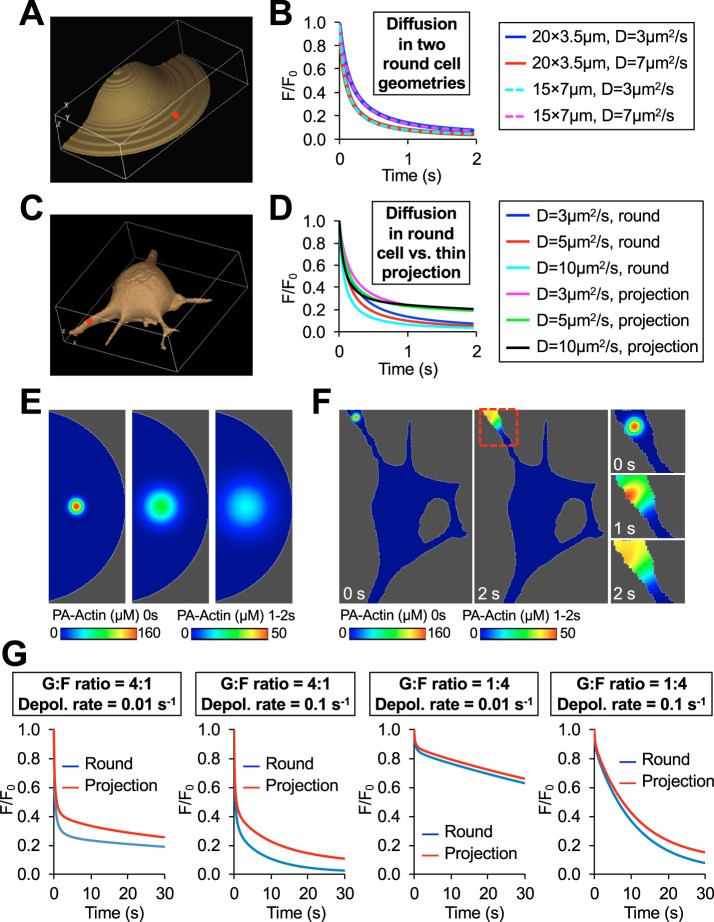
**The significance of cellular geometry in analyzing photoactivation data.** (A) A round cell geometry similar to the one used for MAAP of CAD cells (shown in Fig. 1) except that it is 7 μm tall and has a 15 μm radius. (B) A line graph showing the results of simulations from a diffusion-only version of MAAP in two different round cell geometries (shown in Fig. 1 and Fig. 3A) using two different diffusion rates (annotated D). (C) An amoeboid cell geometry with several long, narrow projections extending from the cell body. The region that was photoactivated in the simulations is shown in red. (D) A line graph showing the results of simulations from a diffusion-only version of MAAP using either the round cell geometry (round) or the geometry containing long thin projections (projection) (shown in Fig. 1 and Fig. 3C) using three different diffusion rates (annotated D). (E) Simulated actin photoactivation in the rounded cell geometry. (F) Simulated actin photoactivation within the geometry of a long narrow projection. A close up of the region outlined in red in the middle panel is shown in the images on the left. For E and F, the concentration of photoactivated actin (PA-actin) is color coded according the scale bars underneath the image. (G) Results of simulations from round and projection geometries (shown in Fig. 1 and Fig. 3C) using the parameters listed above the graph. G-actin:F-actin ratio, G:F ratio; depolymerization rate, Depol. rate.

To determine whether cellular morphology is an important parameter in the analysis and interpretation of photoactivation experiments, we simulated the decay of photoactivated actin intensity in a cell with long narrow protrusions and compared it with two rounded cell geometries that differ in spread area and height (Fig. 4A,C). Under every set of parameters tested, we found no differences between the two round geometries (Fig. 4B), hence small variations in cell height and area in the CAD cells used for photoactivation experiments will not affect the outcome of MAAP analysis. However, there were substantial differences in the decay dynamics between the rounded and long thin protrusive geometries (Fig. 4E–G). Owing to a limited diffusion of actin monomers in the *x*- and *y*-directions in the elongated protrusion, the fluorescence decay rates were slower compared to those in the rounded cell where two-dimensional (2D) diffusion is essentially unrestricted (Fig. 4D). This demonstrates that if FRAP or photoactivation is performed in cells with very different morphologies or where there is a differential restriction in diffusion, the traditional comparison using the decay half-time can lead to an incorrect conclusion. Thus, the accurate fitting of experimental data requires a model that matches the cellular geometry where the experiment was performed.

**Fig. 5.**
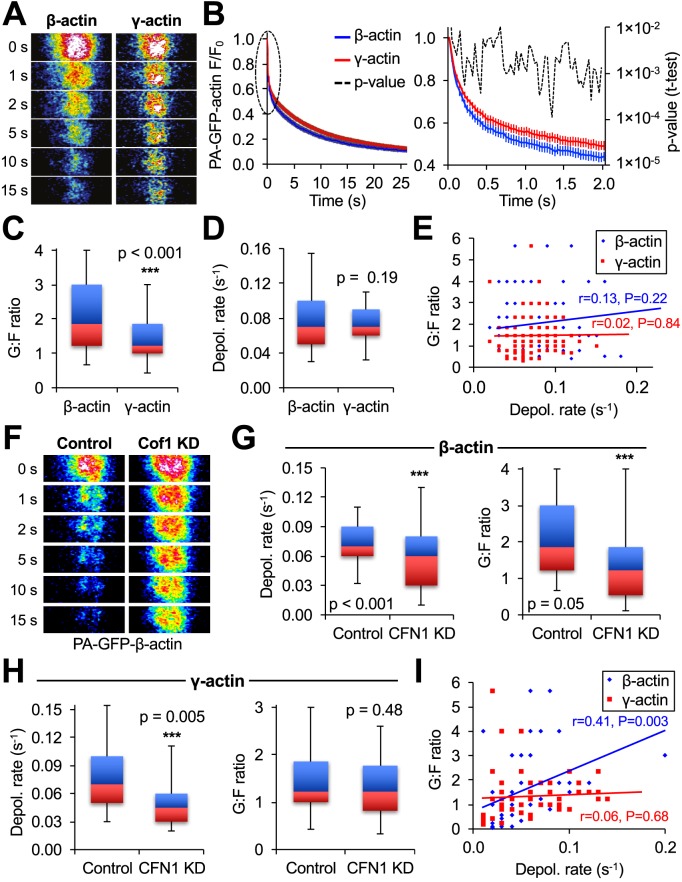
**MAAP analyses of β- and γ-actin dynamics.** (A) Representative photoactivation images from cells expressing either PA-GFP–β-actin or PA-GFP–γ-actin. (B) Mean fluorescence decay curves for PA-GFP–β-actin (*n*=92) and PA-GFP–γ-actin (*n*=105). Error bars are 95% confidence intervals. (C) Box-and-whisker plots showing the distribution of calculated depolymerization rates for PA-GFP–β-actin and PA-GFP–γ-actin from individual experiments. (D) Box-and-whisker plots showing the distribution of the calculated G-actin:F-actin (G:F) ratios of PA-GFP–β-actin and PA-GFP–γ-actin from individual experiments. (E) Scatter plot showing calculated depolymerization rates and G:F ratios from individual experiments. Linear fits of the data and Pearson product-moment correlation coefficients are shown in the same color as the corresponding data set. (F) Representative photoactivation of PA-GFP–β-actin in cells expressing a control scrambled shRNA (Control) or an shRNA knocking down cofilin 1 (Cof1 or CFN1 KD). (G) Box-and-whisker plots showing the distribution of G:F actin ratios and F-actin depolymerization rates of individual decay curves from control (*n*=92) or Cof1 KD (*n*=50) cells expressing PA-GFP–β-actin. Cof1 KD cells exhibited a substantial decrease in both the rate of F-actin depolymerization and the G:F ratio. (H) Box-and-whisker plots showing the distribution of G:F actin ratios and F-actin depolymerization rates of individual decay curves from control (*n*=105) or Cof1 KD (*n*=50) cells expressing PA-GFP–γ-actin. Cof1 KD cells exhibited a substantial decrease in the rate of F-actin depolymerization but not in the G:F ratio. (I) Scatter plot showing calculated depolymerization rates and G:F ratios of PA-GFP–β-actin and PA-GFP–γ-actin in Cof1 KD cells from individual experiments. Linear fits of the data and Pearson product-moment correlation coefficients are shown in the same color as the corresponding data set. In the absence of cofilin 1, the depolymerization rate and G:F ratio became positively correlated for PA-GFP–β-actin but not PA-GFP–γ-actin. Box-and-whisker plots denote the 95th (top whisker), 75th (top edge of box), 25th (bottom edge of box) and 5th (bottom whisker) percentiles, and the median (bold line in box). *P*-values are from a two-tailed Student's *t*-test.

Next, we used MAAP to identify isoform-specific differences in actin dynamics. Previous reports have shown differences in the localization and function of β- and γ-actin (Belyantseva et al., 2009; Bunnell et al., 2011; Perrin and Ervasti, 2010), but much less is known about how the different isoforms behave dynamically at the subcellular level. The traditional analysis of the *t*_1/2_ of the average fluorescence decay curve (1.8 s for PA-GFP–γ-actin and 1.2 s for PA-GFP–β-actin; mean curves shown in Fig. 5B) would suggest that β-actin has faster depolymerization. However, MAAP analysis of individual fluorescence decay curves showed that only one parameter has significant isoform differences, namely the G-actin:F-actin ratio. β-actin was present at higher G-actin:F-actin ratios than γ-actin (Fig. 5B,C), even though the filaments displayed equal depolymerization kinetics (Fig. 5D). The presence of a larger pool of β-actin monomers was confirmed by treating live cells expressing EGFP–actin with saponin and measuring the rate of fluorescence loss (Fig. S2A). Application of 0.02% saponin, a mild detergent, causes actin monomers to leave the cell while actin polymerized into filaments remains inside (Watanabe and Mitchison, 2002). The rate at which EGFP–β-actin left the cells was significantly faster than for EGFP–γ-actin (Fig. S2A), verifying the MAAP result that predicted β-actin is present at the higher monomer-to-filament ratio (Fig. 5C). We also evaluated whether PA-GFP–actin expression levels might influence the outcome of a MAAP analysis, but did not find any relationship between PA-GFP fluorescence intensity and calculation of the G-actin:F-actin ratio or depolymerization rate (Fig. S2B). Interestingly, there was also no correlation between the G-actin:F-actin ratio and depolymerization rate for either isoform (Fig. 5E), indicating that both β- and γ-actin are maintained at sufficiently high monomer concentrations in the cytoplasm that they do not immediately rely on local filament disassembly to replenish their stock.

Cofilin (cofilin 1)-mediated filament severing has been shown to be a major factor in both disassembling actin networks and regulating the amount of G-actin available for polymerization into the lamellipodia of stimulated cells (Breitsprecher et al., 2011; Chin et al., 2016; Elam et al., 2013; Kiuchi et al., 2007; Lappalainen and Drubin, 1997; Vitriol et al., 2013; Wen et al., 2007). Hence, we next tested whether these effects were actin isoform specific. Whereas knockdown of cofilin resulted in a substantial decrease in calculated depolymerization rates for both β- and γ-actin (Fig. 5G,H), only the β-actin G-actin:F-actin ratio was significantly decreased (Fig. 5G). Furthermore, the G-actin:F-actin ratio becames positively correlated with the local depolymerization rate in the absence of cofilin for β- but not γ-actin (Fig. 5I). Taken together, this means that the available pool of β-actin monomers is dependent upon cofilin to remain at a sufficiently high concentration so that it is independent of filament disassembly while the γ-actin pool is cofilin independent. This is of interest because previous work with photoconvertable β-actin has shown that cofilin-mediated filament severing is necessary to create G-actin for increased polymerization into lamellipodia (Kiuchi et al., 2007). The results presented here would suggest that these experiments would not have given the same results if a γ-actin probe was used, assuming that the dynamics of the two isoforms was preserved between the two cell types.

**Fig. 6.**
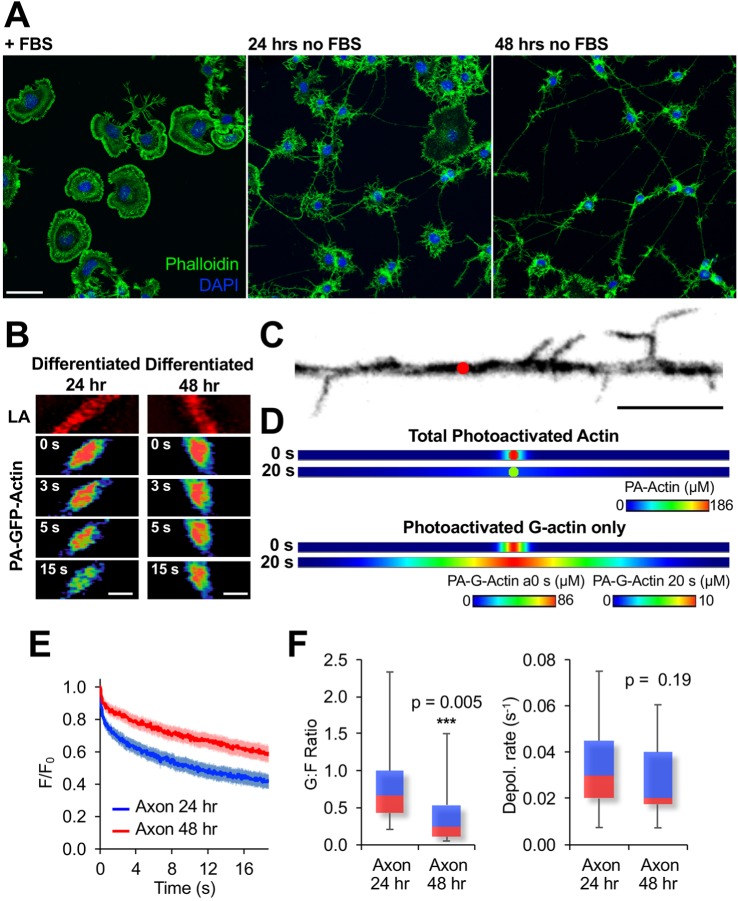
**MAAP analyses of actin dynamics in the axonal projections of differentiating CAD cells.** (A) Example of CAD cell differentiation when cultured in medium lacking fetal bovine serum (FBS). F-actin is stained with phalloidin (green) and nuclei are stained with DAPI (blue). Scale bar: 50 μm. (B) Examples of photoactivation experiments in axons from 24 and 48 h differentiated CAD cells. Lifeact–mRuby (LA) is shown in red in the top panels to highlight the shape of the axon that was photoactivated. (C) A representative example of an axon-like projection from a 48 h differentiated CAD cell with a 1 μm circular region (shown in red) representing where the actin would be photoactivated in this cell. Only regions of the axon that were free of filopodia, 1 μm wide and 20 μm from either the cell body or axonal tip were used for this study. Scale bar: 10 μm. (D) Representative images from the MAAP simulation of axon photoactivation showing the distribution of photoactivated actin at 0 and 20 s after PA-GFP fluorescence was turned on. The top panels show the total actin (F- and G-actin) distribution and the bottom panels show the G-actin distribution only after photoactivation. Images are color coded based on the scale beneath them. The images are scaled identically to in C and the same scale bar can be used. (E) Mean fluorescence decay curves for PA-GFP–actin in axons from 24 (*n*=50) and 48 h (*n*=40) differentiated CAD cells. Error bars are 95% confidence intervals. (F) Box-and-whisker plots showing the distribution of calculated G-actin:F-actin (G:F) ratios and depolymerization rates from individual experiments. The G:F ratio, but not the depolymerization rate, was substantially reduced in 48 h differentiated axons. *P*-values are from two-tailed Student's *t*-test.

To demonstrate the effectivity of MAAP in a completely different geometry than a round spreading cell, we utilized it to analyze actin dynamics in the axon-like projections of differentiating CAD cells. When deprived of serum, CAD cells become increasingly neuron-like, expressing neuron-specific markers and considerably altering their morphology (Qi et al., 1997). The latter includes sending out long thin axonal protrusions (Fig. 6A). We photoactivated actin in axonal protrusions that were 1 µm wide and at least 20 µm from the cell body or protrusion tip (Fig. 6C). Experiments were performed in axonal protrusions at 24 and 48 h post differentiation. We found that actin fluorescence was significantly retained in the photoactivated region in 48 h differentiated axonal protrusions (Fig. 6B). We constructed a MAAP geometry that was 1 µm wide and 40 µm long to mimic the morphology of the axonal protrusions (Fig. 6D). MAAP analysis revealed that the depolymerization rates of filaments from both 24 and 48 h differentiated cells was identical but the G-actin:F-actin ratio was substantially reduced in older axons. Furthermore, as was the case with comparing β- and γ-actin, the difference in the *t*_1/2_ of the 24 and 48 h mean curves (1.52 s for 24 h axons and 2.88 s for 48 h axons), would most likely lead investigators to assume that the slower mobility was due to a decrease in depolymerization. MAAP, which allows us to separately measure G- and F-actin dynamics, revealed that it was only the G-actin:F-actin ratio that was altered was between the two time points. When the G-actin concentration is decreased and depolymerization remains the same it is indicative of increased polymerization and actin network assembly. This might reflect important early steps in the creation of the axon cytoskeletal support network (Ganguly et al., 2015; Xu et al., 2013). This will be an interesting avenue to pursue in greater detail using MAAP because very little is known about actin dynamics within the axon shaft.

## DISCUSSION

We originally used MAAP to calculate the diffusion coefficient of G-actin. While this has been done many times over the years, estimates of actin diffusion have varied over an order magnitude, from 3–30 μm^2^/s, using different cell lines and analysis methods (Kiuchi et al., 2011; McDonald et al., 2006; McGrath et al., 1998; Zicha et al., 2003). The idea for developing MAAP for calculating actin diffusion rates came after we realized that a substantial amount of fluorescence was being lost during and immediately after photoactivation (Fig. S1E), which would cause one to underestimate that amount of G-actin that was present in the photoactivated region and misinterpret the decay curve. 3D simulations would allow us to correct for this fluorescence loss and more accurately predict the actual diffusion rate. It should be noted that fluorescence redistribution during photoactivation and fluorescence loss due to frame acquisition rate occurs even in fast imaging systems specifically designed for FRAP and photoactivation studies. We performed our experiments at 30 frames/s and photoactivation occurs during imaging, not between frames (Fig. S1D), and even then ∼15% of the photoactivated molecules have diffused away before we can capture the first image (Fig. S1D–F). This effect is significantly enhanced if the diffusion rate increases or frame rate decreases (Fig. S1F). Not correcting for fluorescence loss can dramatically change the interpretation of an actin photoactivation experiment. This is illustrated in Fig. S1G, where we compare simulated data where all parameters are identical except the frame acquisition rate. Decreasing the frame rate from 30 frames/s to 4.25 frames/s would cause the same data to have a substantial apparent reduction of actin mobility if both data sets were normalized to the first frame taken after photoactivation. Thus, fluorescence loss during photoactivation and in the interval between image acquisitions is a real-world problem that must be corrected for in order to obtain accurate information from a photoactivation experiment, which can be done with MAAP.

We then created this newer version of MAAP that also distinguishes between fluorescence loss after photoactivation due to G-actin diffusion or F-actin depolymerization. This allowed for the calculation of discrete differences in the cytoplasmic G-actin:F-actin ration for β- and γ-actin (Fig. 5C) and in the axonal G-actin:F-actin of γ-actin at different time points during differentiation (Fig. 6F). Importantly, in both of these instances the F-actin depolymerization rate was identical between the groups being compared. Traditional analysis of the half-time of decay (*t*_1/2_), showing a reduction in mobility through an increase in the *t*_1/2_, would most likely have garnered the opposite interpretation. However, we validated our measurement of the cytoplasmic G-actin:F-actin by treating cells expressing EGFP–actin with saponin and measuring the fluorescence loss. As predicted by MAAP, there was an increased loss of EGFP–β-actin than of EGFP–γ-actin (Fig. 2A). Additionally, after eliminating so many potential variables that could possibly contribute to fluorescence dynamics following actin photoactivation, including fluctuating diffusion rates (Fig. S1C), F-actin movement (Table S1), photobleaching (Materials and Methods), differential expression of PA-GFP–actin (Fig. S2C,D) and variations in cell size or shape (Fig. 4B), the G-actin:F-actin ratio and depolymerization rate are the only logical candidates left to explain the fluorescence loss in their respective portions of the fluorescence decay curves. Furthermore, by generating hundreds of potential outcomes for a photoactivation experiment and having excellent sensitivity between iterations (Figs S3 and S4), MAAP is able to discriminate the precise contributions of F- and G-actin to fluorescence decay after photoactivation. In the future, MAAP can be improved by using gene editing to tag endogenous actin with a photoactivatable fluorescent protein. In doing so, actin dynamics could be measured without worrying about overexpression artifacts or the contribution of unlabeled actin. If the amount of actin was measured in these gene-edited cells, MAAP would be able to calculate exact concentrations of actin monomers and filaments instead of a ratio.

The finding of differentially regulated pools of β- and γ-actin monomers was of interest. Recent work has determined that G-actin-binding proteins help direct monomers toward specific sites of actin assembly, allowing certain types of filaments to recruit actin when the biochemical odds would otherwise be stacked against them (Rotty et al., 2015; Suarez et al., 2015; Vitriol et al., 2015). Isoform selectivity would allow for an additional layer of regulation governing actin polymerization. Whether the monomer was β-actin or γ-actin might change the G-actin-binding protein or type of filament it is most likely to interact with (reviewed in Perrin and Ervasti, 2010), biasing it towards a different type of actin assembly. An example of where this would be relevant is in bundled actin filaments found within a densely branched lamellipodia. The bundled filaments only polymerize from the tip, which would account for relatively few barbed ends compared to the much larger number of barbed ends found at the end of all of the short Arp 2/3 branched filaments they would be surrounded by. In order for the actin to be able to reach the tips of the actin bundles, they would need to be preferentially recruited there. Additionally, we found that that the pool of β-actin monomers was controlled by cofilin-mediated filament depolymerization but the γ-actin pool was not (Fig. 5I) indicating that the β-actin monomer pool is more sensitive to the steady state of lamellipodial turnover (Vitriol et al., 2015). Interestingly, in our previous work, γ-actin was found to specifically localize to the leading edge of actively protruding lamellipodia (Vitriol et al., 2015). This might happen because the lamellipodia needs to recruit additional monomers beyond what is necessary to maintain steady state turnover (Suarez and Kovar, 2016) and to change the composition of the actin network towards more filament subtypes that promote persistent membrane extension. Certainly, the presence of isoform-specific dynamic behavior adds an additional layer of complexity to how polymerizing actin filaments choose the monomers they incorporate from the different subsets of G-actin pools present within a cell.

## MATERIALS AND METHODS

### DNA constructs

The following DNA constructs were used in this study: Lifeact–mRuby (pN1-Lifeact-mRuby), PA-GFP–actin (pC1 PA-GFP-γ-actin, pC1 PA-GFP β-actin) and EGFP–actin (pEGFP-C1 EGFP γ-actin, pEGFP-C1 EGFP β-actin). DNA constructs were prepared using Endotoxin-free Maxi Prep kits (Qiagen).

### CAD cell culture

CAD cells (purchased from Sigma-Aldrich) were cultured in Dulbecco's modified Eagle's medium (DMEM) with F12 (Gibco) supplemented with 8% fetal calf serum (FCS), 1% L-Glutamine and 1% penicillin-streptomycin. CAD cells were differentiated in the same medium without serum. They were imaged in DMEM/F12 without Phenol Red (Gibco) supplemented with 15 mM HEPES. Prior to imaging, CAD cells were plated on coverslips coated with 10 μg/ml laminin (Sigma). After 90 min, normal culture medium was exchanged for imaging medium (DMEM/F12 without Phenol Red + 20 mM HEPES). CAD cells were transfected 12–24 h prior to imaging with the appropriate constructs using the Neon electroporation system (Invitrogen) using a single 1400 v 20 ms pulse. 1 μg of DNA was used for each 10 μl electroporation. This protocol routinely gave >99% transfection efficiency and <10% cell death.

For short hairpin RNA (shRNA)-mediated knockdown, cells were infected with lentiviral particles (Santa Cruz Biotechnology) that expressed both an shRNA hairpin and a gene for puromycin resistance. Infection was performed in the presence of 5 µg/ml polybrene (Santa Cruz Biotechnology). At 48 h after infection, cells were selected with 10 µg/ml puromycin (Santa Cruz Biotechnology). This concentration was chosen because it killed 100% of uninfected cells within 24 h. After selection, cells were continuously cultured in medium containing 10 µg/ml puromycin, except during imaging experiments. Quantification of CFN1 knockdown was as previously described (Vitriol et al., 2013).

CAD cells are a unique mouse neuroblastoma cell line that differentiate into a neuronal-like cell morphology upon serum withdrawal (Qi et al., 1997). We routinely use serum withdrawal to validate CAD cells by ensuring that they were able to undergo neuronal differentiation as evidenced by the formation of long (>100 μm) narrow projections after 2 days.

### Microscopy

Photoactivation experiments were performed on a Nikon A1R laser scanning confocal microscope equipped with an automated *z*-drive with Perfect Focus, multiple laser lines with AOTF control, motorized *x-y* stage, a second resonance scanner for high-speed imaging and photoactivation, a stage incubator with CO_2_ and temperature control (Tokai Hit), and multiple photomultiplier tube detectors. Cells were mounted in a custom live-cell chamber. All experiments were performed using a 60×1.49NA PlanApoN TIRF oil immersion objective. PA-GFP–actin was photoactivated with the resonance scanner of the Nikon A1R microscope, which allows for the simultaneous photoactivation with the 405-nm laser line and monitoring of GFP and mRuby with the 488-nm and 561-nm laser lines, respectively. Photoactivation was conducted with a single 65-ms pulse. To maximize imaging speed, a 55-μm long and 3.5-μm tall rectangular region of the field of view was used for imaging and the scanning direction was set to bidirectional. This allowed an imaging speed at 30 frames/s. The PA ROI was a 2-μm diameter circle for round CAD cells. Photoactivation was performed 20 μm away from the cell edge and in regions where that did not have visible accumulation of F-actin as determined by Lifeact localization. The PA ROI was a 1-μm diameter circle for the axon-like projections of differentiated CAD cells. Experiments in axons were performed at least 20 μm from the cell body or tip of the axon and only in regions that did not have filopodia. Experiments where the axon moved, exhibited a change in morphology or exhibited a large flux of actin moving through it were excluded from the analysis.

### Image analysis

Image analysis was performed with Nikon Elements and software and exported into Microsoft Excel for analysis. Image series for PA-GFP–actin were background subtracted and normalized to the first time point after photoactivation. It was determined that photobleach correction was not necessary by measuring the integrated intensity of the whole cell immediately after photoactivation and then after an experiment had commenced. Both PA-GFP and mRuby fluorescence were within 5% of their initial levels following an experiment.

### Virtual cell modeling

#### Reaction schemes

To simulate experimental results presented in this paper, the BioModel on VCell platform was created. The reaction scheme of Biomodel includes the following species: F-actin and G-actin in both dark and photoactivated states, the laser, and two species, nuc and denuc, that govern the polymerization and depolymerization kinetics. During the photoactivation phase, the species ‘laser’ transforms actin, both free molecules and filamentous into photoactivated ‘PA’ forms of the same species. All ‘PA’ forms were assumed to have the same diffusion coefficient and polymerization and depolymerization rate as non-activated forms.

#### Cell geometry

The experiments with photoactivatable actin were performed on cells adhered to glass substrates and fully spread. To model cell geometry in the numerical simulations, we used the analytical geometry employed in the actin polymerization model (Ditlev et al., 2009; Kapustina et al., 2010) for a rounded cell with two sets of parameters: radius *r*=20 μm, a height (H) in the center of 3.5 μm and thickness at the edge of the lamellipodium of 0.3 μm (Fig. 3), and radius *r*=15 μm, a height (H) in the center of 7 μm and thickness at the edge of the lamellipodium of 0.4 μm. The analytical expression for a cylinder with length of 40 μm (also verified with 80 μm) and photoactivated area with *r*=0.5 μm was used for axon simulation. For protrusive cells, the geometry was derived from a microscope image, which is publicly available from user ‘les’. To decrease the computational time, the model equations for the FDAP simulations in the rounded cell were solved only in half of the cell, because the irradiated area (*r*=1 μm) was substantially smaller than the entire cell body.

#### Photoactivation simulation

The experimental photoactivation measurements were performed using a circular photoactivated region (ROI, *r*=1 μm) on a confocal microscope using a 60×1.49 NA objective with the pinhole completely open. For these parameters, the half width of half maximum of point spread function in the lateral direction is *r*_0_=170 μm and in axial direction is z_0_>3 μm. It was shown previously (Braeckmans et al., 2003) that under these conditions (5 *r*_0_<ROI) we can assume that the ROI is photoactivated uniformly. To mimic the experimental setup in the simulation, we uniformly photoactivated during *t*=65 ms a 2-μm diameter cylinder through the cytoplasm positioned so that its centroid was located 10 μm from the cell center. The parameter characterizing the laser intensity was adjusted to excite 100% of the fluorophores in the region. Each simulation was run for 25 s after photoactivation. We assume that during our experiments the system was in thermodynamic equilibrium and the ratio between F- and G-actin was constant.

#### Simulations

The resultant system of partial differential algebraic equations was solved employing the Fully-Implicit finite volume solver in Virtual Cell using a regular rectangular grid of 201×101×18 elements with a variable time step for photoactivation simulations and 201×201×23 elements with variable time step for simulations of cell center photoactivation. The accuracy of the simulations was checked by refining the mesh. The computational model for simulations are available as the BioModel entitled ‘GF-actin FDAP’ and ‘Axon_FDAP’ in the Virtual Cell database under username ‘marynka’ (http://vcell.org/). The total concentration of actin was kept constant and equal to 200 μm during all simulations. The ratio between F- and G-actin was varied between 1:19 (10 μM of F-actin and 190 μM of G-actin) to 19:1 (190 μM of F-actin and 10 μM of G-actin). For each ratio value, the photoactivation data was simulated using an F-actin depolymerization rate that varied between 0.01 s^−1^ and 0.2 s^−1^ (total twenty simulations for each G-actin:F-actin ratio). After simulation, the average concentration of photoactivated actin molecules in both F- and G- forms within the photoactivatable area was collected, transferred to an Excel table and normalized by the value at 0.035 s after photoactivation, similar to the first value in time obtained experimentally. A custom Matlab script (available on request) was used to determine the best fit, based on the lowest calculated RMSD value, between the experimental and simulated data. For better fitting of the initial part with fast decay due to G-actin diffusion, the first 3 s of the curve had a time step of 0.05 s whereas the rest had a time step of 0.2 s.

#### Sensitivity analysis

To determine how the fluorescence intensity of the photoactivated actin in the ROI is responsive to small changes in the G-actin:F-actin ratio and depolymerization rates (*K*_dep_), we performed a sensitivity analysis. Using our library of computed data for the fluorescence decay after photoactivation, we calculated for each time point the difference in PA-actin concentration between two decay curves that had the same *K*_dep_ but different F-actin concentration. The difference in F-actin between each curve was 10 μM, which is 0.5% of the total actin in the simulation. An example for *K*_dep_=0.07 s^−1^ is presented in Fig. S3. With this analysis, it is apparent that for the slow depolymerization rate, all time points on decay curve except the few at the beginning that are dominated by G-actin diffusion have a similar sensitivity to F-actin concentration with a small maximum near time *t*=3 s after photoactivation. For the faster depolymerization rate, the sensitivity for F-actin concentration between 10 μM and 150 μM reaches a maximum value near 1.5 s, and its value is similar to that of slow depolymerization sensitivity. At later time points the sensitivity to F-actin concentration is reduced and then lost. Not surprisingly though, the decay for high F-actin concentrations (>150 μM) is sensitive to small F-actin changes at all time points.

To see how all F-actin concentrations used in our simulation behave with different *K*_dep_ rates, we averaged all of the values that are presented in each differential curve. This means that each curve in Fig. S3B, for example, after averaging across all of the time points will present only one value. Recalculated values for all curves in the simulation library are presented in Fig. S3C. This analysis shows that decay curves with slower depolymerization rates are more sensitive when the G-actin:F-actin is high. Curves with faster depolymerization rates have higher sensitivity when the G-actin:F-actin is low (*F*>150 μM).

Next, we determined the sensitivity of PA-actin fluorescence intensity values on small differences of depolymerization rate while keeping the G-actin:F-actin ratio constant. Fig. S4 shows an example of this relationship over time for G-actin:F-actin=1 (*F*=100 μM). The dependence is very complex. The different parts of the decay curves have different sensitivity for *K*_dep_ and the sensitivity depends on *K*_dep_ value. As expected, the beginning of the fluorescence decay curves are not sensitive to *K*_dep_ because decay is due purely to diffusion, especially for high G-actin:F-actin ratios. Curves with slower *K*_dep_ rates showed maximum sensitivity near the end of the time course. For curves with fast depolymerization rates (*K*_dep_>0.1 s^−1^), the sensitivity has an earlier maximum followed by a steady decrease. The average sensitivity for all *K*_dep_ values in the simulation to changing G-actin:F-actin ratios (Fig. S4C) was calculated similar to Fig. S3C. The analysis shows that the highest sensitivity occurs when there is a high concentration of F-actin (and thus a small G-actin:F-actin ratio) and the depolymerization rate is slow. The sensitivity of curves with fast depolymerization rates is small and almost independent of the G-actin:F-actin ratio.
